# Construction of A High-Density Genetic Map and Mapping of Fruit Traits in Watermelon (*Citrullus Lanatus* L.) Based on Whole-Genome Resequencing

**DOI:** 10.3390/ijms19103268

**Published:** 2018-10-21

**Authors:** Bingbing Li, Xuqiang Lu, Junling Dou, Ali Aslam, Lei Gao, Shengjie Zhao, Nan He, Wenge Liu

**Affiliations:** Zhengzhou Fruit Research Institute, Chinese Academy of Agricultural Sciences, Zhengzhou 450009, China; bbingli@foxmail.com (B.L.); luxuqiang@caas.cn (X.L.); junlingdou@163.com (J.D.); mianaliaslam538@gmail.com (A.A.); gaolei027@163.com (L.G.); zhaoshengjie@caas.cn (S.Z.); henan@caas.cn (N.H.)

**Keywords:** watermelon, genetic map, WGR, fruit bitterness, rind color, seed coat color

## Abstract

Watermelon (*Citrullus lanatus* L.) is an important horticultural crop that is grown worldwide and has a high economic value. To dissect the loci associated with important horticultural traits and to analyze the genetic and genomic information of this species, a high-density genetic map was constructed based on whole-genome resequencing (WGR), a powerful high-resolution method for single-nucleotide polymorphism (SNP) marker development, genetic map construction, and gene mapping. Resequencing of both parental lines and 126 recombinant inbred lines (RIL) resulted in the detection of 178,762 single-nucleotide polymorphism (SNP) markers in the parental lines at a sequencing depth greater than four-fold. Additionally, 2132 recombination bin markers comprising 103,029 SNP markers were mapped onto 11 linkage groups (LGs). Substantially more SNP markers were mapped to the genetic map compared with other recent studies. The total length of the linkage map was 1508.94 cM, with an average distance of 0.74 cM between adjacent bin markers. Based on this genetic map, one locus for fruit bitterness, one locus for rind color, and one locus for seed coat color with high LOD scores (58.361, 18.353, 26.852) were identified on chromosome 1, chromosome 8, and chromosome 3, respectively. These prominent loci were identified in a region of 6.16 Mb, 2.07 Mb, and 0.37 Mb, respectively. On the basis of current research, the high-density map and mapping results will provide a valuable tool for identifying candidate genes, map-based gene cloning, comparative mapping, and marker-assisted selection (MAS) in watermelon breeding.

## 1. Introduction

Watermelon [*Citrullus lanatus* (Thu nb.) Matsum. & Nakai (2n = 2x = 22)] is an important member of Cucurbitaceae that is commercially produced globally and China is currently both the top producer and consumer of watermelon, with a cultivated area of 189 million ha in 2016 (FAO, http://faostat.fao.org). Watermelon has an approximate genome size of 425 Mb [1,2].

High-density genetic maps are valuable tools for quantitative trait loci (QTL) mapping, gene mapping, and identifying candidate genes. One important prerequisite for constructing a high-density genetic map is the identification of numerous polymorphic markers. Genetic linkage maps were constructed for watermelon in several previous studies. These maps were constructed based on isozymes [3,4], randomly amplified polymorphic DNA (RAPD), restriction fragment length polymorphisms (RFLP), amplified fragment length polymorphisms (AFLP), sequence-related amplified polymorphism (SRAP) markers, and simple sequence repeat (SSR) markers [5,6,7]. However, the maps constructed with these techniques are low-density and have a higher number of linkage groups than anticipated.

In order to produce a more saturated map, the first high-density genetic map was constructed with 698 SSRs, 219 insertion-deletions (InDels) and 36 structural variations (SV) using an F8 population of 103 recombinant inbred lines (RILs). The map consisted of 11 linkage groups and spanned almost 800 cM with a mean marker interval of 0.8 cM [8]. In another study, the first single-nucleotide polymorphism (SNP)-based genetic map for watermelon identified 388 SNP markers was constructed [9,10]. An integrated genetic map was also constructed that contained 698 SSRs, 219 InDels, 36 structural variations, and 386 SNP markers from four maps [11], This integrated map has a much higher marker density than the previously reported genetic map, spanning 798 cM with a mean marker interval of 0.6 cM. Although this integrated map had a higher marker density, it was far from saturated and did not have enough markers to be used for marker-assisted selection (MAS) or for cloning important genes [12].

The development of high-throughput sequencing technology has enabled the study of species in greater detail and the use of this modern molecular method has become a new trend. Next-generation sequencing (NGS) technology makes large scale SNP marker discovery feasible and sequencing a population is a very promising approach to constructing high-density maps for QTL mapping and gene cloning [13]. High-throughput sequencing has been used to produce high-density genetic maps for many crops such as rice [14], soybean [15], melon [16], cabbage [17], and cotton [18]. In recent years, several genetic maps for watermelon has been published on the basis of high-throughput sequencing technology [12,19,20,21]. The marker density for watermelon has been increased and several new QTLs have been detected via these genetic maps. Meanwhile, almost all of the genetic map was constructed by specific length amplified fragment sequencing (SLAF-seq) method. Whole-genome resequencing (WGR) is a sequence-based genotyping method, which is faster and more accurate than marker-based genotyping methods for constructing a genetic map and detecting recombination breakpoints [22]. This mapping method could improve the efficiency of QTL mapping and marker development significantly compared with previous method [23].

Fruit bitterness is a negative agricultural trait, and rind and seed coat color are crucial parameters in the horticulture industry. However, the genetic phenomena and genes associated with these important traits are not well described in watermelon. 

Fruit bitterness trait exists in wild watermelon. To take advantage of the genetic resources of the wild watermelon, breeders sometimes need to cross wild and cultivated watermelon. Fruit bitterness is harmful for breeding work. Cucurbitacins are the chemical compounds responsible for bitterness in cucurbits [24,25]. Morever, recent studies indicated that cucurbitacins are medicinally valuable in the treatment of cancer because they induce cell cycle arrest, apoptosis, and suppress the growth of cancer cells [26,27]. Cucurbitacin research has mainly been focused on cucumber, and there are very few reports of cucurbitacins in watermelon. Genes that influence the bitterness of cucumber fruit were mapped to a 442 kb region on chromosome 5 that contained 67 predicted genes [28]. Shang et al. [29] identified 9 genes in the biosynthetic pathway of cucurbitacin C that contribute to the bitterness of cucumber fruit and discovered the regulator of this pathway, the Bt (bitter fruit) transcription factor. Zhou et al. [30] used comparative genomics among cucurbits to speculate that *ClBt* (*Cla011508*) was a transcription factor that regulated the fruit-specific accumulation of cucurbitacin in watermelon.

Rind color is one of the important agronomic traits in horticulture crops and its visual qualities significantly influence preference of consumer in market. It has been studied in many species such as pepper [31], cucumber [32], and wax gourd [33] among others. However, there have been only a few studies on rind color in watermelon. According to Weetman [34], phenotypes of stripe pattern and rind color were controlled by three alleles located at a single locus. Kumar and Wehner [35] found that two loci controlled the solid dark green rind color. And it was reported that a solid dark green rind color is inherited with light green rind color in a duplicate dominant epistatic fashion [35]. Park et al. [36] constructed a linkage map to find a marker (chr8_26061) closely linked to the depth of rind coloration. Five genes are candidates for controlling yellow and green rind color in watermelon [37].

Seed coat color plays an important role in breeding seeded watermelons and other plants such as sesame, soybean, and pea. Seed coat color is related to the biochemical characteristics of seeds, the amounts and activities of antioxidants within them [38]. However, there have been few studies on seed coat color in watermelon and the genetic mechanism that accounts for it has not been elucidated. McKay [39] suggested that watermelon seed coat color is determined by three genes and Chi et al. [40] identified four loci for seed coat color on chromosome 8. 

In this study, a recombinant inbred line (RIL), containing 126 individuals were developed from a cross between the inbred lines 9904 and Handel. We attempted to use this population to construct a high-density genetic map based on WGR method, which could effectively reduce the time and efforts required to genotype a mapping population [23]. We aimed to identify candidate region for fruit bitterness, rind color, and seed coat color, which can facilitate the identification of candidate genes and marker-assisted selection breeding programs for watermelon.

## 2. Results

### 2.1. Phenotypic Analysis of the Recombinant Inbred Line (RIL) Population

**Figure 1:**
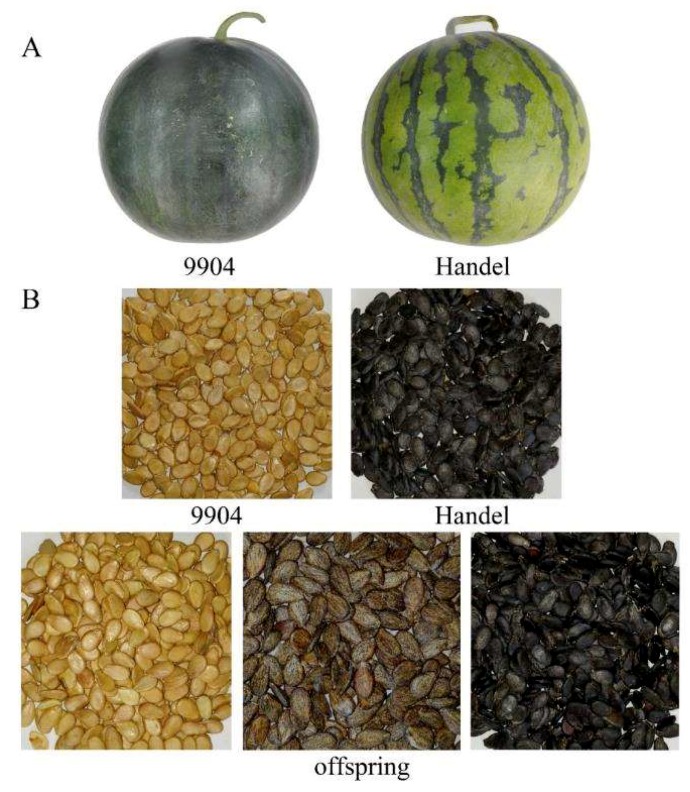
The fruits and seeds of two parent lines. (**A**) the fruits of two parental lines. (**B**) the seeds of two parental lines and recombinant inbred lines (RILs) population.

The RIL population was planted with the parental lines in three environments. It was easy to distinguish the different phenotypes of the mature fruits. Phenotyping results were identified based on the three growth environments, and results were similar in all the environments. The female parent (9904) had bitter fruit, while the fruit from the male parent (Handel) was not bitter. After phenotyping, 68 plants had bitter fruits, and 58 plants had non-bitter fruits in the RIL population. The rind colors of the parents were significantly different, the fruit of the female parent (9904) had a dark green rind, while the fruit of the male parent (Handel) was light green. A total of 56 individuals from the RIL population had fruits with dark green rinds, and 70 individuals had fruits with light green rinds. Seed coat color between the parents was obviously different. The male parent (Handel) had black seeds, and the female parent (9904) had light yellow seeds. There were three phenotypes in the RIL population; 29 individuals had black seed coats, 60 individuals had light yellow seed coats, and 37 plants had mottled seed coats (Figure 1).

### 2.2. Analysis of Whole-Genome Resequencing (WGR) Data and Single-Nucleotide Polymorphism (SNP) Markers

Whole-genome resequencing of the RIL population and both parental inbred lines produced a total of 7.67 Gbp and 8.81 Gbp of high-quality reads for the 9904 and Handel inbred lines, respectively. A total of 177.08 Gbp of high-quality reads were obtained from the 126 individuals in the RIL population. The average Q30 ratio was more than 85%, and the average GC content was nearly 35% for the RIL individuals (Table S1). The distribution of SNP mutations and coverage of assembly scaffolds by high-quality reads indicated that the genome resequencing was sufficiently random (Table S1, Figures S1 and S2). The average coverage depths of the markers were 19-fold for the male parent, 17-fold for the female parent, and 2.99-fold for their progeny (Table S1).

A total of 178,762 SNPs with at least a four-fold sequencing depth were obtained by analyzing the parental lines. All of the SNP sites in the RILs were integrated into a recombination bin unit, and 2132 recombinant bins comprising 103,029 SNPs were used to construct the genetic map (Table 1, Tables S2 and S3). To our knowledge, the SNP markers which were mapped are much more numerous than recently reported watermelon genetic maps.

### 2.3. Construction of the Genetic Map

**Figure 2:**
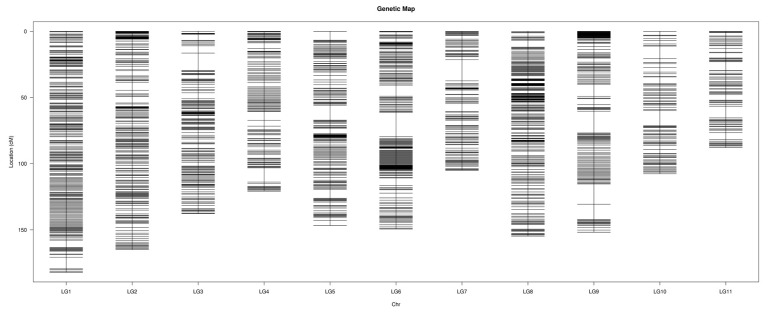
High-density genetic map of watermelon constructed by bin markers.

The final high-density genetic map consisted of 11 chromosomes and was based on 2132 recombination bins containing 103,029 SNPs with a well-distributed linkage distance (Figure 2). The total length was 1508.94 cM, with an average distance of 0.74 cM between adjacent bin markers.

In general, the bin markers were well-distributed on the genome and approximately 98.63% of the intervals between adjacent markers were less than 5 cM. The largest gap occurred in LG06 and was 18.45 cM. LG1 was the largest LG, which covered 182.08 cM and had 294 bin markers with an average distance of 0.62 cM between adjacent markers. The smallest LG was LG11, which covered 87.88 cM and had 99 bin markers with an average distance of 0.89 cM between adjacent markers (Table 1). 

**Figure 3:**
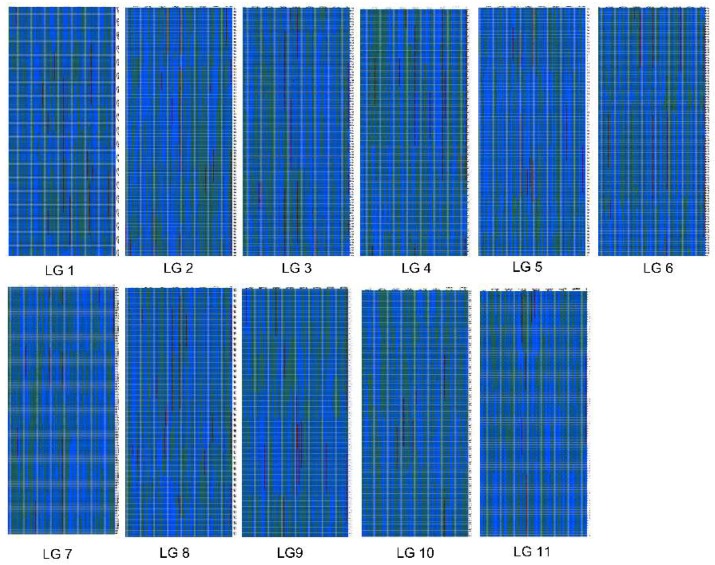
The haplotype map of the genetic map. The x axis represents the markers and the y axis represents 126 samples. Green represents “9904” (the female parent), blue represents “Handel” (the male parent), red indicates heterozygosity, and gray indicates missing data. The same column where the color changed is the location of the recombination events.

Haplotype and heat maps were used to assess the quality of our genetic map. The haplotype map reflected the double crossovers of the population and deletions, which indicated recombination events and marker ordering errors. Haplotype maps were generated for each of the 126 lines in the RIL population as well as the parental lines. As illustrated in Figure 3, almost all of the recombination blocks were clearly defined. 

**Figure 4:**
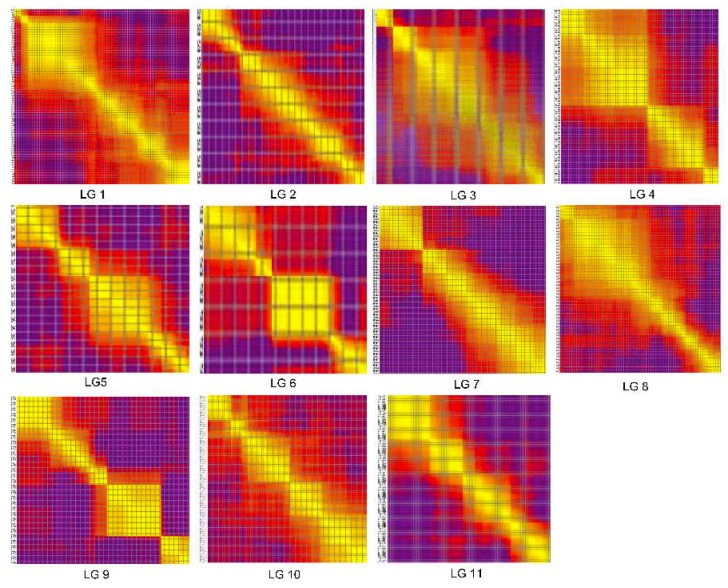
The heat map of genetic map. The x and y axis represent markers. The order of markers on line and row were arranged according to their genetic distance. Each cell represents the recombination rate of two markers. Yellow indicates a lower recombination rate and purple a higher one.

Heat maps of the 11 LGs were generated separately based on pair-wise recombination values for the 2132 recombination bin markers. The linkage relation heat map illustrates the relationship between recombination markers on one chromosome and this can be used to identify potential marker ordering errors. As shown in Figure 4, the closer the distance between different markers, the lower the recombination rate (indicated by the color yellow). Otherwise, a higher recombination rate is indicated by the color purple. These heat maps generally indicated that the construction of our genetic map was accurate, since the linkage groups were easily visualized.

### 2.4. Collinearity of the Genetic and Physical Maps

**Figure 5:**
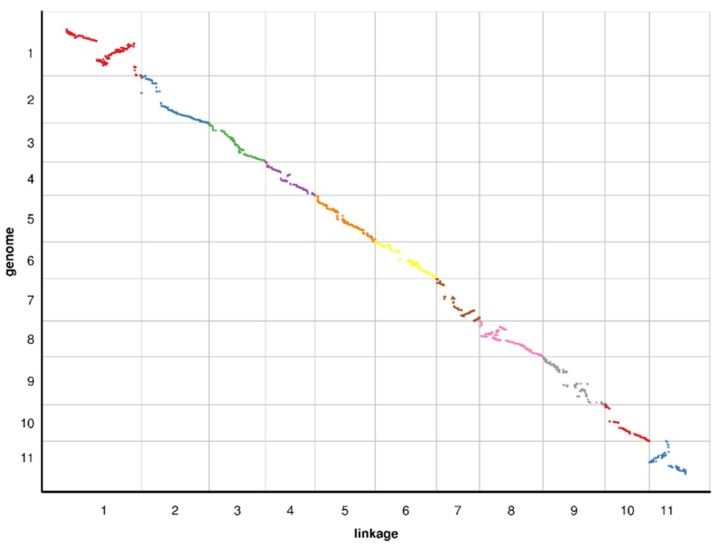
Relationship between genetic and physical positions with each chromosome. In each plot, genetic distance is on the x-axis, and physical distance is on the y-axis.

To evaluate the collinearity between the genetic map and the watermelon reference genome (97103), all bin markers were mapped to the watermelon reference genome. As shown in Figure 5, the relationships between the genetic and physical maps were generally linear for 11 chromosomes except for chromosome 1 and chromosome 11. Marker order was reversed between the genetic and physical maps in the regions spanning from 73.548 cM to 164.140 cM on LG1 and from 0 cM to 46.950 cM on LG11. This result was consistent with previous studies that found an inconsistency between the genetic and physical positions on the map for a segment on chromosome 1 and 11 [12,20,41,42]. A part of the region on chromosome 7 (53.857–105.013 cM) and chromosome 8 (0–65.391 cM) were not properly represented on the physical map that were consistent with previous reports [20,21]. The Spearman correlation coefficient of each LG is shown in Table 2. The average Spearman correlation coefficient between the genetic and physical positions of all LGs is 0.94. All of our results showed that these LGs have high levels of genetic collinearity with the physical map. The high collinearity suggested that the markers accurately cover 11 chromosomes and that they sufficiently cover the watermelon genome.

### 2.5. Detection of Candidate Regions Associated with Fruit Traits

**Figure 6:**
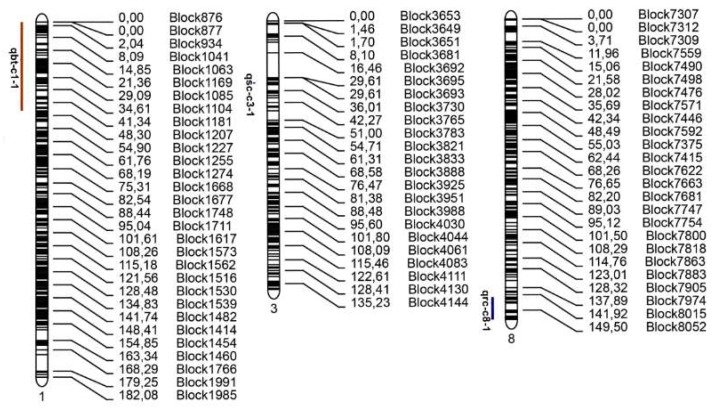
Mapping results for fruit bitterness, seed coat color, rind color on watermelon linkage groups 1, 3 and 8. Bin marker names and map distance in centimorgans (cM) are shown on the right side of each linkage group.

The interval mapping (IM) method was used to identify loci that were significantly associated with fruit bitterness and rind color [43,44]. Composite interval mapping (CIM) was used to identify loci for seed coat color [43,45]. One locus for fruit bitterness, one locus for rind color, and one locus for seed coat color were detected on chromosome 1, chromosome 8, and chromosome 3 with maximum LOD scores of 58.361, 18.353, and 26.852, respectively (Figure 6, Table 3).

#### 2.5.1. Identification of Candidate Region Associated with Fruit Bitterness

One locus associated with watermelon fruit bitterness were identified on chromosome 1 (Figure 6). The prominent locus with the highest LOD score (58.361) was designated as qbt-c1-1, explained 82.927% of phenotypic variation and displayed a negative additive effect of −0.465. It was identified in the 45.91 cM/6.16 Mb region spanning from block876–block1188. (Table 3, Table S4).

#### 2.5.2. Identification of Candidate Region Associated with Rind Color

We detected one prominent locus on chromosome 8 that was associated with rind color and had a high LOD score of 18.353, which we designated as qrc-c8-1 (Figure 6, Table 3). This locus explained 49.942% of phenotypic variation and was identified in a 12.012 cM/2.07 Mb region. This locus occurred in the marker interval from block8000 to block8110 and displayed a negative additive effect of −0.358 (Table 3, Table S4).

#### 2.5.3. Identification of Candidate Region Associated with Seed Coat Color

One prominent locus associated with seed color was mapped to chromosome 3 (Figure 6). This locus was designated as qsc-c3-1, explained 58.685% of the phenotypic variation and had a high LOD score of 26.852. It was identified in a 0.8 cM/0.37 Mb region and had a negative additive effect of −0.625 (Table 3, Table S4).

### 2.6. Annotation of Candidate Genes

A total of 586 candidate genes were identified in the confidence intervals of the locus associated with fruit bitterness. A total of 576 of the 586 candidate genes had annotation information in the watermelon reference genome, among which 240, 499, 120, 408, and 575 had annotation information in COG, GO, KEGG, Swissprot, and Nr databases, respectively (Table S5). Our analysis using the COG database placed 25 genes in the transcription category; 34 genes in the replication, recombination, and repair category; and 31 genes in the carbohydrate transport and metabolism category. Analysis of the genes in the candidate intervals using the KEGG database identified 36 genes in the metabolic pathways category and 14 genes in the biosynthesis of secondary metabolites category. Using the Nr database, 513 genes were identified from *Cucumis sativus* (Table S6).

A total of 259 genes were identified in the confidence interval of the locus associated with rind color. A total of 258 candidate genes had annotation information, among which 80, 226, 61, 191, and 258 had annotation information in the COG, GO, KEGG, Swissprot and Nr databases, respectively (Table S5). Analysis using the COG database identified 13 genes in the transcription category, 9 genes in the replication, recombination, and repair category, and 13 genes in the signal transduction mechanisms category. Based on KEGG annotations, 18 and six genes were assigned to the metabolic pathways and the biosynthesis of secondary metabolites categories, respectively. A total of 227 genes from *Cucumis sativus* were identified in the Nr database (Table S7).

There were 30 genes identified in the confidence interval of the locus associated with seed coat color. A total of 29 candidate genes had annotation information, among which 6, 25, 5, 19, and 29 had annotation information in the COG, GO, KEGG, Swissprot, and Nr databases, respectively (Table S5). COG analysis identified two genes in the transcription category; three genes in the replication, recombination, and repair category; and two genes in the signal transduction mechanisms category. Using the KEGG database, three genes were identified in the metabolic pathways category. A total of 25 genes were identified from *Cucumis sativus* in the Nr database (Table S8).

## 3. Discussion

### 3.1. Characteristics of the WGR Approach for Developing Markers and Candidate Region Analysis

Genetic maps are important for finding loci of interest, fine mapping, map-based cloning, and marker-assisted breeding [20,33]. The utility of genetic linkage maps depends mainly on the types and numbers of polymorphic markers used to construct the genetic map. Several genetic maps have been constructed for watermelon based on isozymes, RAPD, RFLP, AFLP, SRAP, and STS [5,6,7]. The limited quantity and quality of available markers restricted the development of high-density genetic linkage maps for watermelon. Long-term selection and cultivation for desirable horticultural traits led to a narrowing of the genetic base in cultivated watermelon [46]. The narrow genetic base results in fewer polymorphisms that can be used as conventional markers, which makes it harder to construct a high-density genetic map for watermelon.

The rapid development of next-generation sequencing (NGS) technologies and the publication of the watermelon reference genome sequence made it possible to construct a high-density genetic map using single-nucleotide polymorphism (SNP) markers and accurate genotyping [47]. The NGS-based method has been applied successfully in several species [48,49]. There have been several NGS-based genetic maps published for watermelon in recent years [12,19,20,21]. However, there are very few reports of watermelon genetic maps based on whole-genome resequencing (WGR) [21,40]. This sequence-based genotyping method was faster and more accurate than marker-based genotyping methods for constructing a genetic map and detecting recombination breakpoints [22]. The WGR mapping method could significantly improve the efficiency of QTL mapping and marker development [23]. Based on WGR method, 103,029 SNP markers have been mapped on the genetic map. Many more SNP markers were mapped to the genetic map than previous studies in watermelon [11,12,20,50], which will provide valuable tools for future candidate gene identification, map-based gene cloning, and marker-assisted selection.

In contrast to another genetic map based on the WGR method [21], we not only sequenced both parental lines at a high coverage depth but also sequenced the offspring at a low coverage depth. Our study reports a high-density genetic map based on whole genome resequencing of both parental lines and a RIL population. A prominent feature of the WGR method for constructing genetic maps that was utilized in this research is that it integrated SNP discovery, SNP validation, and genotyping [51]. Sustained progress in high-throughput sequencing has promoted the development of SNP discovery and genotyping analyses tools. Whole-genome resequencing-based methods have been used to construct genetic linkage maps for several species [16,23,51]. We can verify the proper alignment and ordering of sequenced tags by comparison to the reference genome, which can help to impute low-coverage data [52]. The primary advantage of the WGR method for QTL mapping is that recombination breakpoints in each RIL can be inferred. In this study, we sequenced parental lines with a 20-fold sequencing depth and each RIL with 3.32-fold sequencing depth, which was sufficient to detect the recombination breakpoints. We obtained 7.67 Gb, 8.81 Gb, and 177.80 Gb of high-quality reads from the female parent, the male parent, and their progeny, respectively. Recombination intervals that differed from other studies’ genotypic data were eliminated by using strict analysis criteria, and bin markers that were based on accurate genotypic data were used to construct the high-density genetic map. After the genotyping analysis, a total of 2132 recombination bin markers representing 103,029 SNP markers were mapped onto 11 linkage groups. Our result show that the WGR strategy is a powerful method for marker discovery and high-density linkage map construction. Using the WGR mapping method, we captured a large amount of gnome-wide SNPs, which precisely reflect the characteristics of genomic and genetic diversity in watermelon.

### 3.2. Features of the High-Density Genetic Map of Watermelon

In recent years, several genetic maps based on high-throughput sequencing technology have been constructed for watermelon. Ren et al. [12] published a high-density genetic linkage map of watermelon based on the DArT-seq™ method. The map contained a total of 1161 bin markers spanning a total length of 1099.2 cM, with an average distance of 1.0 cM between adjacent bin markers. Cheng et al. [19] used high-throughput re-sequencing to construct an genetic map of watermelon that spanned a total length of 1244.5 cM and contained 125 polymorphic markers consisting of 82 CAPS and 43 SSR. A high-density genetic map was also constructed using large-scale SNP discovery by specific length amplified fragment sequencing (SLAF-seq) [20]. The SLAF-seq-based map contains 2634 SNPs distributed among 11 linkage groups (LGs) spanning 1906.31 cM, with an average distance of 0.72 cM between adjacent markers. Liu et al. [21] used whole-genome resequencing of an F2 population to construct a CAPS-based genetic linkage map that spanned 1836.51 cM, had 11 linkage groups, and contained 301 markers (264 CAPS, 37 SSR).

In the present study, the mapping population was generated from a cross between 9904 and Handel. Handel is an American inbred line, while 9904 is derived from the cross between Chinese cultivar JW and wild egusi seeded watermelon (*Citrullus lanatus* subsp. mucosospermus). The wide genetic diversity between the two parental lines brought a higher frequency of polymorphisms for map construction and significant differences in several traits. A total of 178,763 SNP markers with at least a four-fold sequencing depth were detected in the parental lines based on whole-genome resequencing. All of the SNPs were used to genotype the RIL population with strict filtering rules. 17,366 SNPs, which did not pass the chi-square test (*p* < 0.001), were excluded. The chi-square test efficiently removed false SNPs caused by misalignment of paralogous sequences or sequencing errors. Finally, a total of 2132 recombination bin markers representing 103,029 SNP markers were mapped onto 11 linkage groups. The total length of the linkage map was 1508.94 cM, with an average distance of 0.74 cM between adjacent bin markers. Many more SNP markers were mapped to our genetic map. Recently reported watermelon genetic maps only contained 378 [50], 386 [11], 3465 [12], and 2634 SNP markers [20].

The collinearity of the genetic and physical maps was generally uniform for each linkage group despite the reversal of 90.59 cM and 46.95 cM regions in LG1 and LG11, which was consistent with previous reports [12,20,41,42]. This inconsistency could be due to incorrect assembly of the reference genome, genetic rearrangement or assembly errors [53]. Visual evaluation of the haplotype and heat maps of the genetic map suggested that the RILs were suitable for genetic analysis and that our map was accurately constructed.

This high-density genetic map provides a foundation for the fine mapping of candidate genes, map-based cloning, and MAS-based breeding programs for important agronomic traits.

### 3.3. The Identification of Loci of Fruit Bitterness, Rind and Seed Coat Color

Cucurbitacins, which are the chemical compounds that contribute to bitterness in cucurbits [24,25], are potentially medicinally valuable in the treatment of cancer because they induce cell cycle arrest, apoptosis, and suppress the growth of cancer cells [26,27]. Very few studies have investigated fruit bitterness in watermelon. A fruit bitterness gene was previously mapped to a 442 kb region on chromosome 5 in cucumber that harbors 67 predicted genes [28,29] identified nine cucumber genes in the cucurbitacin C biosynthetic pathway that contributed to bitterness in cucumber fruit and discovered the Bt (bitter fruit) transcription factor that regulates this pathway. Zhou et al. [30] used comparative genomics among cucurbits to speculate that *ClBt* (*Cla011508*) is a fruit-specific transcription factor that regulates cucurbitacin in watermelon. In our study, we identified one prominent loci on chromosome 1. The prominent locus with the highest LOD score (58.361) explained 82.927% of phenotypic variation in a region of 6.16 Mb/45.91 cM. *ClBt* lies within this candidate region. The consistency of these results suggests that fine mapping can be confidently performed.

Rind color is an important trait for breeding watermelons that has been studied in many plants. Huh et al. [31] identified a candidate gene that may impart red and orange colors in pepper. Liu et al. [32] identified a gene associated with white rind in cucumber in a 33 kb region on chromosome 3. A single locus on chromosome 5 was implicated in the control of rind color in wax gourd [33]. A major locus associated with rind color was identified on chromosome 8 in pear [54]. However, there have only been a few reports investigating rind color in watermelon. In a previous report, a marker (chr8_26061) located on chromosome 8 was found to be closely associated with the depth of rind color [36]. Five genes (*Cla002942 Cla004992 Cla009181 Cla017341 Cla018352*) were identified as candidates that may contribute to the yellow and green rind color in watermelon [37]. Kumar and Wehner [35] reported that solid dark green rind is inherited with light green rind in a duplicate dominant epistatic fashion. In our study, we used a RIL population to map the candidate region for dark green and light green rind colors. One prominent locus associated with rind color that had a high LOD score of 18.353 explained 49.942% of the phenotypic variation and was identified in a 12.012 cM/2.07 Mb region on chromosome 8. The marker (chr8_26061) reportedly [36] linked to the depth of rind color was in this candidate region suggesting that the result is very credible.

Seed coat color is an important trait for seeded watermelon because it influences consumer preference. QTLs associated with seed coat color have been reported in other plants. Badani et al. [55] identified three QTLs for yellow seed color in oilseed rape. Wang et al. [56] identified nine QTLs for seed coat color in sesame using a high-density genetic map. There have been fewer studies on seed coat color in watermelon and its genetic mechanism is not clear. McKay [39] reported that watermelon seed coat color was determined by three genes, but Chi et al. [40] identified four loci for seed coat color on chromosome 8. In our study, one prominent locus associated with seed color was mapped to a 0.8 cM/100 kb region on chromosome 3. This locus explained 58.685% of the phenotypic variation with a very high LOD score of 26.852. It could be very useful for fine mapping and future molecular marker-based breeding strategies.

### 3.4. Preliminary Analysis of the Potential Functions of Candidate Genes

A total of 586 candidate genes were identified in the fruit bitterness candidate regions, and these were classified into at least one category in the COG, GO, KEGG, Swissprot, and Nr databases (Table S6). We performed a preliminary analysis of the candidate genes based on their functional annotation. Cucurbitadienol needs to be modified by cytochrome P-450 enzymes and an acyltransferase to form cucurbitacin [29]. Annotations in the Nr database suggested that *Cla011436*, *Cla004922*, and *Cla003641* are related to cytochrome p450, and *Cla011537* is related to an acyltransferase. There is a possibility that these genes contribute to fruit bitterness along with *Cla011508*, which is located near the markers between block1002 and block1032 with high LOD values (Table S2). *Cla011508* is annotated as a bHLH126 GN transcription factor in the Swissprot database. Zhou et al. [30] used comparative genomics among cucurbits to speculate that *ClBt* (*Cla011508*) was a transcription factor that regulated the fruit-specific accumulation of cucurbitacin in watermelon. There is a strong possibility that this transcription factor is related to fruit bitterness as et al. [30] speculated. 

For rind color, 258 candidate genes had annotation information in the candidate region (Table S7). The phenotype of fruit rind color was detected on the basis of chlorophyll content and other pigments [57]. Analysis using GO annotations suggested that *Cla022573* functions in pigment binding, 56 genes play a role in chloroplast functions, and six genes (*Cla022675*, *Cla022530*, *Cla022517*, *Cla022495*, *Cla022718*, and *Cla022725*) have roles in anthocyanin metabolic processes. KEGG analysis predicted that *Cla022543* and *Cla022670* are associated with porphyrin and chlorophyll metabolism and that *Cla022574* is involved in carotenoid biosynthesis. The functions of these genes may contribute to rind color.

For seed coat color, 29 candidate genes had annotation information in the candidate region (Table S8). Various pigments including flavonols, proanthocyanidin, and possibly other phenolic derivatives influence seed coat color. Polyphenol oxidase functions in the biosynthesis of lignin, melanin, and proanthocyanidin [56]. There are accumulated anthocyanins in the epidermal layer of the seed coat for black seeds compared with yellow seeds [58]. According to the GO annotations, there were five genes (*Cla019482*, *Cla019485*, *Cla019486*, *Cla019483*, and *Cla019481*) involved in pigment biosynthetic processes. Analysis using the Nr database showed that *Cla019482*, *Cla019485*, *Cla019486*, *Cla019483*, and *Cla019481* were related to polyphenol oxidase. These genes could contribute to the differences in the seed coat colors among watermelon.

In this study, genes were located on stable candidate region with a high LOD score. Since there is no direct evidence proving that these genes control these traits, further experiments are needed for fine mapping and functional validation of the genes that they contain. The present high-density map and mapping results analysis will provide valuable tools for future candidate gene identification, map-based gene cloning, comparative mapping, and marker-assisted selection (MAS) breeding programs for watermelon.

## 4. Materials and Methods

### 4.1. Plant Material and DNA Extraction

The mapping population consisted of 126 recombinant inbred lines (RILs, F7) and was generated from a cross between 9904 (the female parent) and Handel (the male parent). Handel and 9904 are inbred lines that differ significantly in several traits (Figure 1). The 9904 line was derived from crossing a Chinese cultivar JW and a wild egusi seeded watermelon (*Citrullus lanatus* subsp. mucosospermus). Handel was an inbred line from USA. The high genetic diversity between the two parental lines contributed to a higher frequency of polymorphisms that could be used for map construction and significant differences in several traits including fruit bitterness, rind color and seed coat color. The fruit of 9904 was bitter with a dark green rind and light yellow seed coats, while the fruit of Handel was not bitter and had a light green rind and black seed coats.

The 126 F7 RILs were grown together with parental lines at two locations under three environments in Hainan and Henan province: Sanya experimental station in 2016 (open field, Hainan, China) and Xinxiang experimental station in 2017 (greenhouse and open field, Henan, China).

Young healthy leaves from the two parents and 126 RILs were collected and stored at −80 °C until their DNA was extracted. The genomic DNA was extracted by the Cetyltrimethyl ammonium bromide (CTAB) method [59]. DNA was quantified with a NanDrop-1000 spectrophotometer (NanoDrop, Wilmington, DE, USA) and was evaluated by electrophoresis in a 1.0% agarose gel.

### 4.2. Evaluation of Phenotypes

One fruit per plant was harvested 35 days after self-pollination. All of the traits could be easily discerned in mature fruit. Each fruit was cut lengthwise, photographed, and sampled to evaluate the following traits. (1) Fruit bitterness was characterized as described by Zhang et al. [60]. Briefly, a small piece of flesh from mature fruit was tasted for bitterness by three people who were sensitive to bitterness, and the fruit samples were divided into bitter and non-bitter groups. (2) Rind color was determined by visual observation, and watermelons were divided into light green and dark green groups based on their appearance. (3) Seed coat color was also determined by visual observation, and seeds were divided into light yellow, black or mottled groups.

### 4.3. Sequencing Library Construction and High-Throughput Sequencing

The quality of DNA samples was checked, and the samples were randomly cleaved into 200–500-bp fragments by sonication. The sequencing libraries were constructed by terminal repair, followed by the addition of a 3′ A and a sequencing linker; the samples were then purified and amplified by PCR. The HiSeq2500 system (Illumina, San Diego, CA, USA) was used for paired-end sequencing.

### 4.4. SNP Identification and Genotyping

Raw reads from the HiSeq2500 system (Illumina) were filtered to obtain high-quality reads, and the reads were re-mapped to the reference genome. The program used to filter reads is in house Perl script from Biomarker Technology Co. Ltd. (Shunyi, Beijing, China). By first deleting the reads with adapter and then reads which contain N, more than 10% had been filtered. Remoting the reads that the base (Q ≤ 10) is more than 50%. To analyze the genomic variation, the reads from each sample were counted, and the coverage ratio and distribution of the reads in the 97103 watermelon reference genome (ftp://www.icugi.org/pub/genome/watermelon/97103/v1/) were calculated using the Burrows–Wheeler Aligner program [61]. The “CollectInsertSizeMetric” function in the Picard software toolkit (http://broadinstitute.github.io/picard/) was used to analyze the insert size. The GATK software toolkit 3.8 (Cambridge, MA, USA) was used to detect potential SNPs among the lines [62]. The Mark Duplicate tool was used to eliminate duplicated reads that may have resulted from PCR amplification. GATK was used for base recalibration, variant calling, and to strictly filter the SNPs to get the final SNP clusters. The parameters for the SNP calling and the mapping were default for the parental line and the RILs population. The SNPs identified between the parents were considered polymorphic in the subsequent bin calling. To guarantee the quality of the genetic map, SNPs were filtered: (1) Selecting the markers that were homozygous and incongruous in parental line; (2) the depth of the SNPs from parental line was more than four-fold. Gene genotyping of the progenies were based on parental genotype. The parental line with high sequencing depth could guarantee that genotyping of the progenies is right. Only biallelic SNPs were retained in the final SNP dataset. The SNPs whose segregation patterns were aa × bb were used to construct the genetic map. The missing values were filled with ab genotype. The average sequencing depth was 20-fold in the parents and 3.32-fold in the progenies. A chi-square test was conducted for bin calling, and SNPs that significantly deviated from an extreme segregation distortion (*p* < 0.001) were excluded.

### 4.5. Genetic Map Construction and Evaluation

HighMap software (http://highmap.biomarker.com.cn/) was used to perform linkage grouping, marker ordering, genotyping error correction, and for mapping evaluation. A modified slide window method was used for bin calling [63]. A 1-SNP and a 15-SNP sliding window was used when scanning the chromosomes and for calling the genotype in each window. When the ratio of aa to bb was more than 11 in the sliding window, the segregation pattern was aa; otherwise, the segregation was bb. The SNPs between the adjacent recombinant breakpoints were combined into a recombination bin. The bin markers were used to construct linkage groups with HighMap [64]. The SMOOTH error correction strategy was performed according to the parental contribution of the genotypes [65], and the Kosambi mapping function was used to estimate the map distances. Then, the ALLMAPS program was used to construct the chromosomes by comparing the constructed genetic map and the watermelon genome map [66]. Haplotype and heat maps were constructed using the “draw haplotype-map.pl” and “draw heatmap.pl” Perl scripts respectively.

### 4.6. Relationship between the Genetic and Physical Maps

All of the sequences of the bin markers that were used to construct the linkage map were aligned to the physical sequences of the 97103 watermelon reference genome. Collinearity between genetic and physical positions was determined by plotting genetic marker positions (in centimorgans) against their physical positions (in megabases), and the BLAST program was used to confirm their physical positions in the genome [47]. Spearman correlation coefficients were calculated to assess the collinearity between the genetic and physical maps.

### 4.7. Gene Mapping and Candidate Genes Prediction

The composite interval mapping (CIM) [67] function of MapQTL [68] was used to identify loci related to seed coat color. An association analysis between markers and the fruit bitterness and rind color traits was conducted using the interval mapping (IM) method [44]. LOD thresholds for determining significant loci were estimated from 1000 permutations [69]. This test, which is based on a multiple linear regression model, ensures that the probability of committing a type I error for loci mapped onto the whole genome is less than 5% [70]. A minimum LOD score of 2.5 was used to judge the presence of a loci.

Based on the positions of the flanking markers, all of the genes within the confidence interval were identified as candidates. The candidate genes in the target loci region were analyzed using the annotations from the 97103 watermelon reference genome. Annotations from the GO, KEGG, COG, Swissprot, and Nr databases were used to categorize the candidate genes.

### 4.8. Candidate Regions Naming

Candidate regions were named according to the trait and linkage group location as described by Sun et al. [71]: q + trait abbreviation + chromosome/linkage groups + loci number. The loci for the same trait across different generations and environments were considered common loci when their confidence intervals overlapped.

## 5. Conclusion

In this study, we constructed a high-density genetic map of watermelon with an RIL population using bin markers derived from WGR. The high-density map had a total of 2132 bin markers representing 103,029 SNP markers. Many more SNP markers were mapped to the genetic map in our study than in recently reported studies in watermelon. The total length of the linkage map was 1508.94 cM, with an average distance of 0.74 cM between adjacent bin markers. The results of haplotype and heat maps generally indicated that almost all of the recombination blocks were clearly defined and that our map was accurately constructed. Moreover, we located one locus for fruit bitterness, one locus for rind color, and one locus for seed coat color with high LOD scores. These prominent loci will be analyzed in detail to identify candidate genes. The present high-density map and mapping results will provide valuable tools for future candidate gene identification, map-based gene cloning, comparative mapping, and marker-assisted selection (MAS).

## Figures and Tables

**Table 1:** Distribution of genetic markers on the high-density genetic map.

LGs	Total Bin Markers	Total Distance (cM)	Average Distance (cM)	Max Gap (cM)	Gap < 5 cM (%)
LG01	294	182.08	0.62	8.49	99.32%
LG02	229	165.00	0.72	6.10	99.56%
LG03	218	137.08	0.63	13.15	99.08%
LG04	137	120.79	0.88	10.74	98.53%
LG05	230	146.77	0.64	11.24	98.69%
LG06	216	149.42	0.69	18.45	98.60%
LG07	150	105.01	0.70	16.03	98.66%
LG08	266	154.74	0.58	5.04	99.62%
LG09	179	151.82	0.85	16.43	97.75%
LG10	114	107.63	0.94	11.52	98.23%
LG11	99	87.88	0.89	8.74	96.94%
Total	2132	1508.94	0.74		98.63%

**Table 2:** The Spearman correlation coefficients between the genetic and physical positions of each linkage group (LG).

LG ID	Spearman
LG01	0.8017
LG02	0.9950
LG03	0.9948
LG04	0.9834
LG05	0.9976
LG06	0.9867
LG07	0.8905
LG08	0.8722
LG09	0.9822
LG10	0.9990
LG11	0.8002

**Table 3:** Loci associated with fruit bitterness, rind color, and seed coat color.

Name	Chromosome	Marker Interval	Position (cM)	LOD	PVE	ADD
qbt-c1-1	1	Block876–Block1188	0–45.91	58.361	82.927	−0.465
qrc-c8-1	8	Block8000–Block8110	142.728–154.742	18.353	49.942	−0.358
qsc-c3-1	3	Block3708–Block3722	31.705–32.505	26.852	58.685	−0.625

## Supplementary Materials

Supplementary materials can be found at http://www.mdpi.com/1422-0067/19/10/3268/s1.

## Abbreviations

WGR	Whole-genome resequencing
SNP	Single-nucleotide polymorphism
RIL	Recombinant inbred lines
LGs	Linkage groups
MAS	Marker-assisted selection
QTL	Quantitative trait loci
NGS	Next-generation sequencing
