# Supplementary material for: Construction of A High-Density Genetic Map and Mapping of Fruit Traits in Watermelon (Citrullus Lanatus L.) Based on Whole-Genome Resequencing
Source: Int J Mol Sci. 2018 Oct 21;19(10):3268. doi: 10.3390/ijms19103268 (PMC6214002; doi:10.3390/ijms19103268)
Supplement: Supplementary file 1 [file ijms-19-03268-s001.zip › ijms-365276 suppl/ijms-365276 supp..pdf]

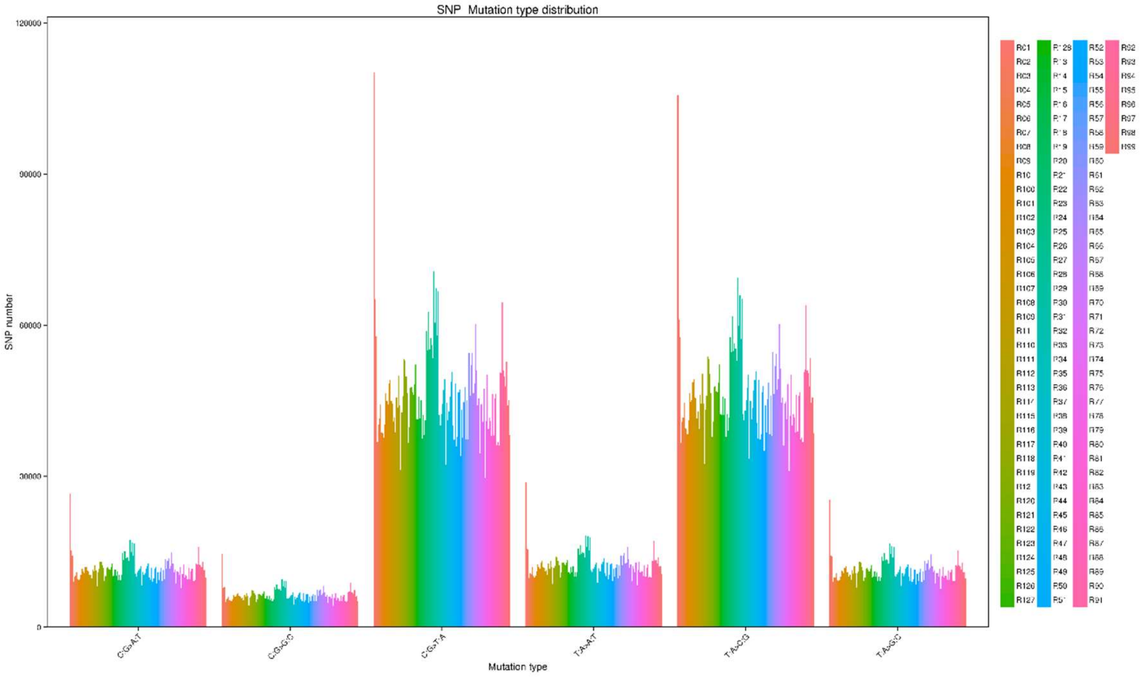

Figure S1. Distribution of SNP quality in resequenced lines. R1 and R2 represent female and male parents, respectively. R3-R128 represents the individuals of the RILs population.

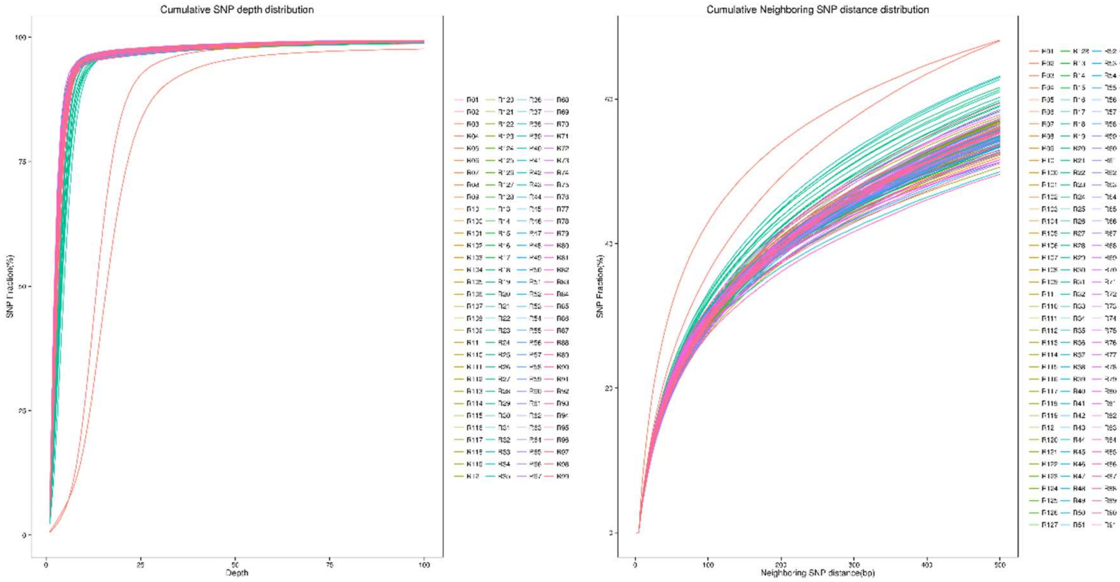

Figure S2. Distribution of SNP mutation types in resequenced lines. R1 and R2 represent female and male parents, respectively. R3-R128 represents the individuals of the RILs population.

Table S1. Results of the resequencing of parents and 126 RILs population.

Table S2. The physical distance of the blocks in the genetic map.

Table S3. The genotype of all the markers mapped on the map. R1 and R2 represent female and male parents, respectively. R3-R128 represents the individuals of the RILs population.

Table S4. The LOD, additive effect, and PVE score of all the markers in the genetic map.

Table S5. The number of the candidate genes in the confidence intervals of the loci in different database analysis.

Table S6. The GO, KEGG, COG, Swissprot, Nr annotation results of the candidate genes in the confidence intervals of the locus of fruit bitterness.

Table S7. The GO, KEGG, COG, Swissprot, Nr annotation results of the candidate genes in the confidence intervals of the locus of rind color.

Table S8. The GO, KEGG, COG, Swissprot, Nr annotation results of the candidate genes in the confidence intervals of the locus of seed coat color.
